# Severe birth defects in children perinatal exposed to HIV from a “real-world” setting: Infectious Diseases National Institute, Bucharest, Romania

**DOI:** 10.7448/IAS.17.4.19699

**Published:** 2014-11-02

**Authors:** Ana Maria Tudor

**Affiliations:** Paediatric Department, Matei Bals Inf Dis Nat Inst, Carol Davila University for Medicine and Pharmacy, Bucharest, Romania

## Abstract

**Introduction:**

The shift in epidemic trends in recent years in Romania shows new problems in regard of HIV vertical transmission, firstly in intravenous drug user's mothers co-infected with hepatitis viruses and with social problems, and secondly the children of young mothers with an old HIV infection and long antiretroviral therapy history.

**Materials and Methods:**

We studied all HIV perinatal exposed children routinely followed up in the Paediatric Department of the National Institute of Infectious Diseases, since January 1st 2006 till December 31st 2012. The analyses consisted of describing the birth defects and association with certain risk factors: gender, mother's age at birth and exposure to antiretrovirals in the first trimester of pregnancy.

**Results:**

We analyzed 244 children born to HIV-infected mothers. The incidence of HIV infection was 16.39%. The rate of birth defects was 39.34% (96/244 cases). The most frequent findings were cardiac malformations (47/96), followed by musculoskeletal defects (24/96), neurologic defects (20/96), urogenital malformations (13/96), digestive tract defects (3/93), metabolic disorders (2/96) and genetic disorders (2/96). We found nine cases of severe congenital anomalies: complex heart defect, total congenital aganglionic megacolon, anal imperforation, Dandy-Walker syndrome, gangliosidosis, Niemann-Pick syndrome, Down syndrome, true hermaphroditism and cleft palate. Two children died during first year of life due to severe malformations. 9% of cases had associated malformations. The gender rate was in favour of males in group with birth defects (58/38) and with no birth defects (82/66). The median age at birth in mothers was 22 years, similar in both groups. The highest mean age at birth was in offspring's mothers with neurologic congenital defects 25, 15 years old, but is not statistically significant (p=0.1). In the studied period the highest number of birth defects were found in 2012, 37 children, compared with less than 15 in previous years (not statistically significant, p=0.07). In our studied patients the risk of birth defects was not statistically associated with HIV transmission or with exposure to antiretrovirals before and in first trimester of pregnancy (p=0.88).

**Conclusion:**

The rate of birth defects among HIV-exposed children was not significantly associated with antiretroviral exposure, but we identify very rare and severe congenital conditions. We have noticed also a trend to increasing number of birth defects in 2012 among studied patients compared to previous years.

